# Grain‐Boundary‐Enriched Cocatalyst Coupled With CuSCN Hole Transport Layer for Efficient Solar Water Oxidation in BiVO_4_ Photoanode

**DOI:** 10.1002/advs.77764

**Published:** 2026-09-16

**Authors:** Wei Zhao, Yaorong He, Xiao Wang, Lin Zhu, Tong Su, Yujie Zhou, Xiang Li, Peiyao Du, Xiaoquan Lu

**Affiliations:** ^1^ College of Chemistry & Pharmacy Northwest A&F University Yangling Shaanxi P. R. China; ^2^ State Key Laboratory of Fluorine & Nitrogen Chemicals School of Chemical Engineering and Technology Xi'an Jiaotong University Xi'an Shaanxi P. R. China; ^3^ Key Laboratory of Bioelectrochemistry and Environmental Analysis of Gansu Province College of Chemistry and Chemical Engineering Key Laboratory of Water Environment Protection in Plateau Intersection (Ministry of Education) Northwest Normal University Lanzhou Gansu P. R. China

**Keywords:** bismuth vanadate, grain boundary, hole transfer layer, photoelectrochemical water splitting, scanning photoelectrochemical microscopy

## Abstract

Solar‐driven photoelectrochemical (PEC) water splitting represents a promising pathway toward carbon neutrality, yet the practical conversion efficiency remains severely constrained by the substantial charge recombination losses. Herein, CuSCN as hole transport layer (HTL) coupled with a grain‐boundary‐enriched NiFe cocatalyst (NiFe‐GB) was integrated with BiVO_4_ photoanodes to improve hole extraction/transport and interfacial oxygen evolution reaction (OER) kinetics. The optimized NiFe‐GB/CuSCN/BVO photoanode delivered a photocurrent density of 6.54 mA cm^−2^ at 1.23 V versus RHE. Through a multi‐technique approach encompassing intensity‐modulated photocurrent spectroscopy (IMPS), Kelvin probe force microscopy (KPFM), scanning photoelectrochemical microscopy (SPECM), we demonstrate markedly improved charge separation, interfacial hole transfer, and surface charge utilization in the integrated architecture. Comparative experiments with NiFe LDH/CuSCN/BVO and trend‐level density functional theory calculations suggest that the grain‐boundary‐enriched NiFe structure modulates electronic states and lowers the energy barrier for OER intermediate transformation. This work highlights cocatalyst microstructure regulation as a complementary strategy to hole transport layer engineering for designing efficient BiVO_4_‐based photoanodes.

## Graphical abstract

A grain‐boundary‐enriched NiFe cocatalyst is coupled with a CuSCN hole transport layer on BiVO_4_ photoanodes. The integrated architecture promotes hole extraction, interfacial charge transfer, and surface OER kinetics, highlighting cocatalyst microstructure regulation as a complementary strategy to hole transport layer engineering.

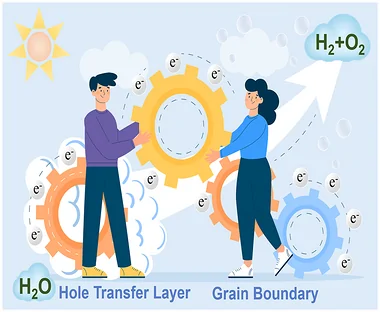

## 1 Introduction

Solar‐driven photoelectrochemical (PEC) water splitting represents one of the most prospective technologies for solar‐to‐hydrogen conversion, and the key to its practical application lies in the development of low‐cost, stable, and high‐performance photoanodes [1, 2, 3]. Among diverse photoanode candidates, bismuth vanadate (BiVO_4_, denoted as BVO) has garnered considerable attention on account of its appropriate visible‐light‐responsive bandgap (∼2.4 eV) and thermodynamically favorable valence band position for water oxidation. Nevertheless, intrinsic bottlenecks are still encountered in BVO, including severe bulk recombination caused by its low hole mobility (≤10^−4^ cm^2^ V^−1^ s^−1^), as well as interfacial charge accumulation associated with sluggish oxygen evolution reaction (OER) kinetics [4, 5]. These coupled challenges limit the achievable photocurrent density, underscoring the demand for synergistic strategies to regulate charge separation, interfacial charge transfer, and surface reaction kinetics in BVO‐based photoanodes [6].

Interfacial engineering has been recognized as a powerful approach for optimizing the performance of photoanodes in PEC water splitting systems [7]. Among the diverse array of approaches, inorganic hole transport layers (HTLs) have proven remarkable efficacy in boosting charge separation efficiency and charge transport dynamics [8, 9]. Copper(I) thiocyanate (CuSCN) stands out as a particularly promising HTL material owing to its remarkable combination of properties, including a wide bandgap (3.4–3.9 eV) for high optical transparency, remarkable hole mobility, high thermal stability, and low cost [10]. Previous studies have shown that CuSCN boosts charge separation and transport efficiency via the formation of energetically favorable interfacial contacts, while enhancing the stability of BVO photoanodes [11, 12]. However, CuSCN intrinsically lacks sufficient catalytic activity for the OER. Accordingly, although CuSCN can promote hole extraction from BVO, the efficient consumption of surface holes still necessitates an appropriate OER cocatalyst.

To accelerate surface OER kinetics, NiFe‐based oxides and hydroxides have been widely employed as cocatalysts owing to their low cost, high activity, and stability in alkaline electrolytes [13, 14, 15, 16]. In addition to composition, the microstructure of NiFe cocatalysts can also influence their catalytic behavior. In electrocatalytic systems, grain boundaries (GBs) are generally regarded as defect‐rich regions that can regulate local electronic structures, modulate the adsorption of oxygen‐containing intermediates, and promote catalytic transformations [17, 18, 19]. However, whether such beneficial grain boundary effects can be translated to PEC photoanodes remains insufficiently understood, providing the direct motivation for this investigation. This issue is particularly important for semiconductor/HTL/cocatalyst architectures, where photogenerated holes must be generated in BVO, extracted and transported through the HTL, and finally consumed at the cocatalyst/electrolyte interface. It should be noted that while CuSCN/BiVO_4_ photoanodes and NiFe‐modified BiVO_4_ photoanodes have been investigated separately in prior studies, the central question we address is how a grain‐boundary‐enriched NiFe cocatalyst behaves at such multi‐component photoelectrocatalytic interfaces and how its functionality couples with CuSCN‐mediated hole transport to improve interfacial charge utilization and OER kinetics.

In this study, a grain‐boundary‐enriched NiFe cocatalyst modified CuSCN/BVO photoanode, denoted as NiFe‐GB/CuSCN/BVO, was designed to construct a coupled hole extraction–transport–surface consumption pathway, achieving a photocurrent density of 6.54 mA cm^−2^ at 1.23 V vs. RHE. In this architecture, CuSCN serves as a hole transport layer to facilitate hole extraction from BVO, while NiFe‐GB acts as a microstructure‐regulated OER cocatalyst to promote interfacial hole utilization. By comparing NiFe‐GB/CuSCN/BVO with conventional NiFe LDH/CuSCN/BVO, we further clarify the beneficial role of grain‐boundary‐rich NiFe structures in modulating OER kinetics at the photoelectrocatalytic interface. This work highlights cocatalyst microstructure regulation as a complementary design strategy to hole transport layer engineering for efficient BiVO_4_‐based photoanodes.

## 2 Results and Discussion

**FIGURE 1:**
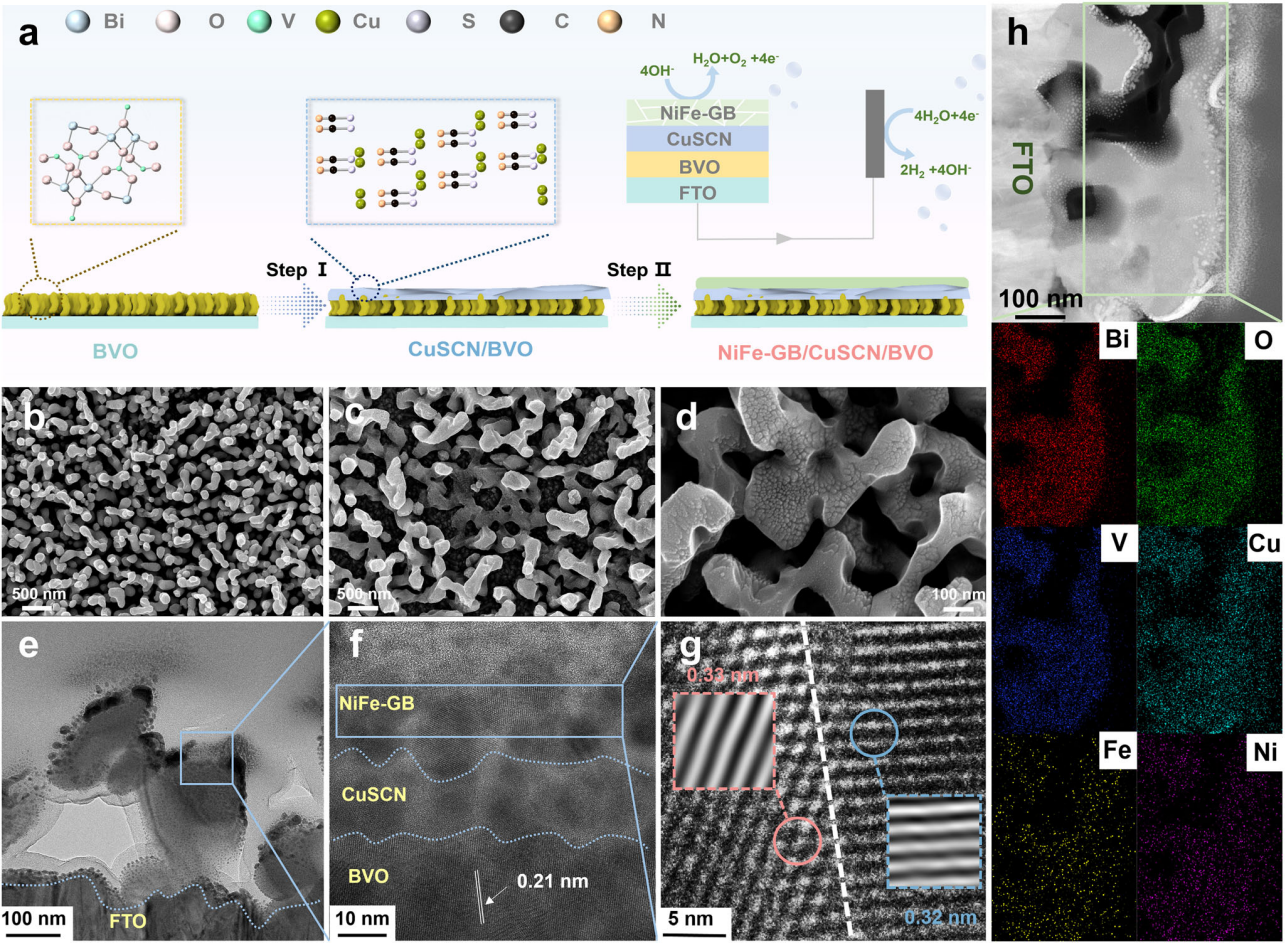
(a) Schematic representation of the fabrication procedure for the photoanode. (b–d) SEM images of (b) bare BVO, (c) CuSCN/BVO, and (d) NiFe‐GB/CuSCN/BVO photoanodes. (e) FIB‐prepared TEM sample. (f) HR‐TEM image of the NiFe‐GB/CuSCN/BVO. (g) Magnified HR‐TEM image highlighting the NiFe‐GB related region. (h) Cross‐sectional EDS elemental mapping of FIB‐prepared NiFe‐GB/CuSCN/BVO.

Figure 1a schematically outlines the fabrication process of NiFe‐GB/CuSCN/BVO photoanode. Following previously reported method, a nanoporous BVO photoanode was first prepared on a fluorine‐doped tin oxide (FTO) glass substrate [20, 21]. Subsequently, a CuSCN hole transport layer was deposited onto the BVO surface via spin‐coating. Finally, a grain‐boundary‐enriched NiFe catalyst (denoted as NiFe‐GB) was synthesized using an economical droplet immersion method at room temperature, and then coated onto the CuSCN‐modified BVO substrate to achieve a NiFe‐GB/CuSCN/BVO integrated system.

Scanning electron microscopy (SEM) of bare BVO shows a homogeneous distribution of worm‐like nanostructures with diameters measuring 150 ∼ 300 nm across the FTO surface (Figure 1b). After spin‐coating the CuSCN precursor solution, the BVO underwent a morphological transformation, with the gaps between adjacent nanorods becoming filled with CuSCN (Figure 1c). Furthermore, nanoparticles were observed on the surface following NiFe‐GB deposition (Figure 1d). As shown in Figure 1e, the focused ion beam (FIB)‐processed sample exhibits a distinct three‐layer structure. The BVO substrate in Figure 1f further reveals well‐defined lattice fringes with an interplanar spacing of 0.21 nm, indexed to the (051) plane of monoclinic BVO [20]. Moreover, as observed in the magnified high‐resolution transmission electron microscopy (HR‐TEM) image (Figure 1g), the NiFe‐GB region exhibits a highly polycrystalline nature, characterized by a prominent grain boundary (marked by a white dashed line). The adjacent nanodomains display interplanar spacings of 0.33 and 0.32 nm. Such localized lattice mismatch and distinct spatial misorientation robustly indicate the formation of boundary‐rich microstructures. The coexistence and spatial distribution of Fe and Ni within the NiFe‐GB cocatalyst is corroborated by the FIB‐prepared cross‐sectional energy‐dispersive spectrometry (EDS) elemental mapping (Figure 1h) and XPS signals (Figure S5). Figure S1 further confirms the introduction of a high density of grain boundaries into NiFe‐GB. The formation of nanograins is likely attributable to the Ni^2+^‐induced weakly acidic environment, which suppresses the extensive crystallization of metal hydroxides [22].

The X‐ray diffraction (XRD) patterns (Figure S2) of BVO, CuSCN/BVO and NiFe‐GB/CuSCN/BVO exhibit diffraction peaks that match well with the monoclinic BVO phase (JCPDS PDF#14‐0688) and the conductive FTO substrate [23]. The lack of any discernible variations in the XRD patterns of CuSCN/BVO and NiFe‐GB/CuSCN/BVO, when compared to bare BVO, may be due to too few of CuSCN and NiFe‐GB catalyst to be recognized [24]. Additionally, several typical characteristic peaks at 127, 213, 328, 370, and 829 cm^−1^ are observed in the Raman spectrum (Figure S3), which can be attributed to the vibrational modes of BVO [25]. No changes in the characteristic peaks after the deposition of the CuSCN and NiFe‐GB, suggesting that the introduction of these layers does not alter the internal atomic structure of the BVO substrate [12].

The full X‐ray photoelectron spectroscopy (XPS) survey spectrum (Figure S4) of NiFe‐GB/CuSCN/BVO photoanode confirms the presence of all expected elements, which is consistent with the EDS results. Notably, positive binding energy shifts are observed for the Bi 4f and V 2p core levels in both CuSCN/BVO and NiFe‐GB/CuSCN/BVO compared to pristine BVO (Figure S5a,c). And, a slight positive shift in the Cu 2p binding energy following the deposition of NiFe‐GB (Figure S5d). In the high‐resolution O 1s spectra (Figure S5b), the main low binding energy component can be assigned to lattice oxygen (O_L_) in BVO, while the higher binding energy components are generally associated with surface oxygen defect related species (O_surf_), hydroxyl groups, and adsorbed oxygen species (O_ads_) [26, 27]. After the sequential deposition of CuSCN and NiFe‐GB, the relative intensity of the higher binding energy O 1s component changes. Finally, the chemical states of Fe and Ni in the NiFe‐GB cocatalyst were verified. The high‐resolution Fe 2p XPS spectrum (Figure S5e) exhibits two main peaks located at 710.9 and 724.3 eV, corresponding to the Fe 2p_3/2_ and Fe 2p_1/2_ core levels, respectively [28]. Figure S5f displays the high‐resolution Ni 2p XPS spectrum, showing peaks at 855.6 eV (Ni 2p_3/2_) and 873.1 eV (Ni 2p_1/2_), together with their corresponding satellite peaks, which collectively confirm the presence of Ni^2+^ [29]. As shown in Figure S6, CuSCN/BVO exhibits a distinct N 1s signal at approximately 398.04 eV, which is assigned to the –S–C≡N group of thiocyanate in CuSCN [12]. After deposition of NiFe‐GB, the N 1s signal becomes weaker and broader, likely because the CuSCN layer is partially covered by the NiFe‐GB cocatalyst and the detectable CuSCN signal is attenuated due to the limited probing depth of XPS.

**FIGURE 2:**
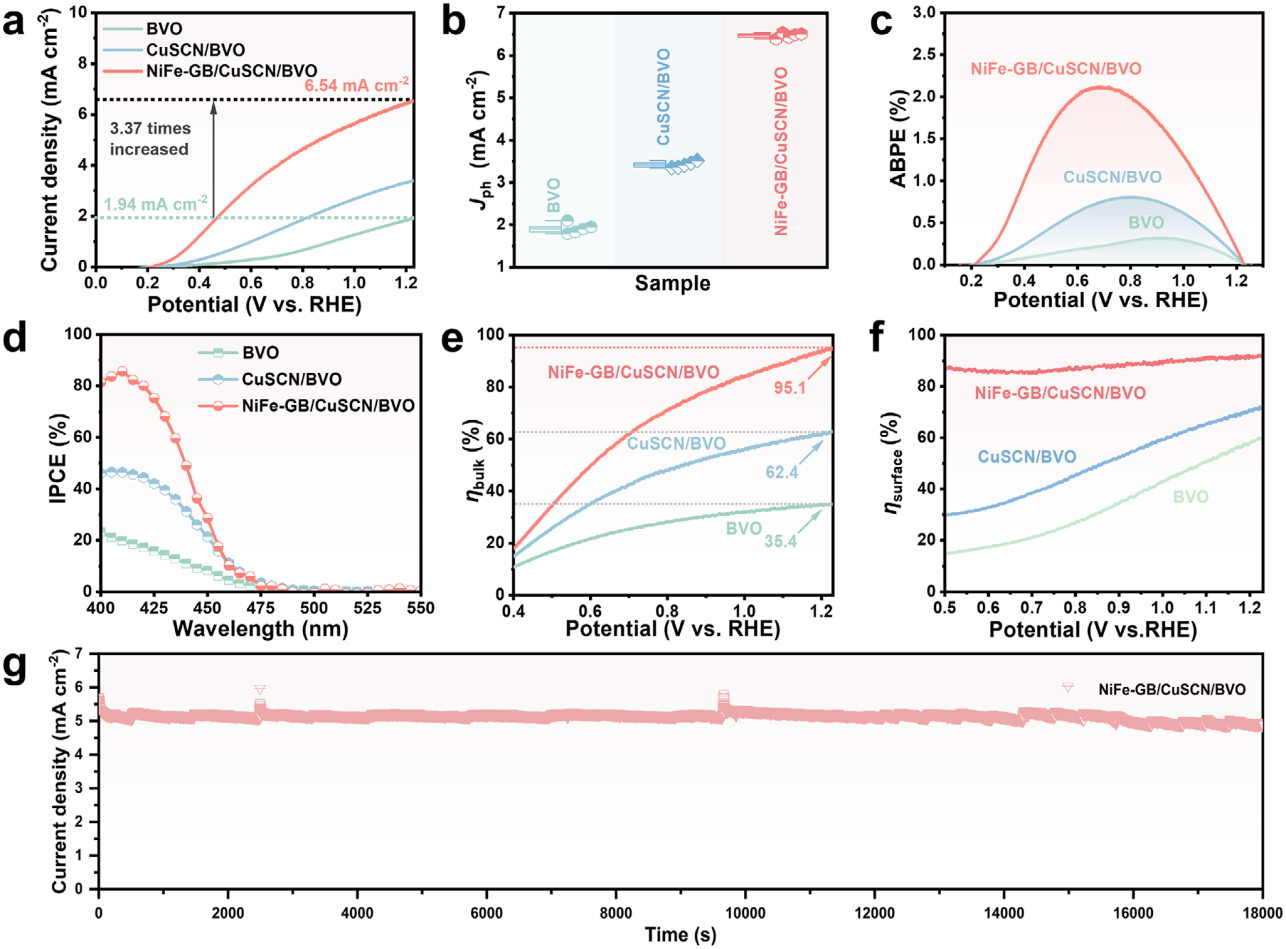
(a) LSV curves measured under AM 1.5G illumination. (b) Statistical analysis of water oxidation photocurrent density at 1.23 V versus RHE based on independently prepared photoanodes (*n* = 5). Individual data points are shown. The box plot displays the minimum, maximum, and mean values. (c) ABPE curves. (d) IPCE spectra. (e) Bulk charge separation efficiency (*η*_bulk_) and (f) surface charge injection efficiency (*η*_surface_) of different photoanodes. (g) Chronoamperometric stability test of the NiFe‐GB/CuSCN/BVO photoanode at 1.0 V versus RHE.

The PEC water splitting performances of the fabricated photoanodes were evaluated in a 1.0 m KBi buffer electrolyte (pH = 9.5), with illumination provided by an AM 1.5 G solar simulator (100 mW cm^−2^). The pristine BVO photoanode exhibits limited activity with a photocurrent density of only 1.94 mA cm^−2^ at 1.23 V vs. RHE, which is attributable to severe charge recombination and sluggish OER kinetics [30, 31]. The modification with a CuSCN hole transport layer (CuSCN/BVO) substantially improves the performance, yielding a photocurrent density of 3.39 mA cm^−2^. Remarkably, the grain‐boundary‐engineered NiFe‐GB/CuSCN/BVO photoanode delivers an impressive photocurrent density of 6.54 mA cm^−2^ at 1.23 V vs RHE, surpassing not only the control samples in this study but also the performance of most state‐of‐the‐art BVO‐based photoanodes documented in recent literature (Figure 2a and Table S1) [31, 32, 33, 34, 35, 36, 37, 38, 39, 40, 41, 42, 43, 44, 45, 46, 47, 48, 49, 50]. To quantitatively deconvolute the synergistic contributions of the CuSCN hole transport layer and the NiFe‐GB cocatalyst, the water oxidation photocurrent at 1.23 V vs RHE was statistically analyzed over multiple independent measurements (Figure 2b, Figure S7 and Table S2). The consistent and stepwise performance enhancement observed across all photoanodes from pristine BVO to CuSCN/BVO and then to NiFe‐GB/CuSCN/BVO, demonstrates the exceptional reproducibility of our fabrication protocol. This trend also supports that the improved performance of the final architecture is associated with the synergistic integration of CuSCN and NiFe‐GB, rather than random experimental variation.

The grain boundary‐engineered NiFe‐GB/CuSCN/BVO photoanode achieves a maximum applied bias photon‐to‐current efficiency (ABPE) of 2.11% at 0.69 V vs. RHE (Figure 2c). This efficiency is notably superior to those of the CuSCN/BVO counterpart (0.81% at 0.80 V vs. RHE) and pristine BVO photoanode (0.32% at 0.92 V vs. RHE). Moreover, the NiFe‐GB/CuSCN/BVO photoanode exhibits a maximum incident photon‐to‐current efficiency (IPCE) of 85.3% at 410 nm (Figure 2d). The bulk charge separation efficiency (*η*_bulk_) and surface charge injection efficiency (*η*_surface_) were quantified using sodium sulfite (Na_2_SO_3_) as a hole scavenger (Figure 2e,f and Figure S8). While the deposition of CuSCN on BVO leads to a modest improvement in *η*_surface_, its primary contribution is a significant boost in *η*_bulk_, increasing from 35.4% for pristine BVO to 62.4% for CuSCN/BVO at 1.23 V vs RHE. Notably, the subsequent introduction of the NiFe‐GB cocatalyst further improves both efficiencies, increasing *η*_bulk_ to 95.1% and markedly enhancing *η*_surface_. This result indicates that NiFe‐GB not only acts as a superior OER catalyst but also collaborates with the CuSCN layer to improve the overall charge separation process. The NiFe‐GB/CuSCN/BVO also displays good durability, retaining over 85% of its initial photocurrent during a 18 000 s test at 1.0 V vs RHE (Figure 2g). Post‐stability SEM characterization supports the structural stability of the photoelectrode (Figure S9), revealing that the NiFe‐GB/CuSCN/BVO retains a worm‐like structure with nanoparticle‐loaded surface, while the XRD patterns (Figure S10) show no detectable changes in phase or crystallinity. Gas evolution measurements for NiFe‐GB/CuSCN/BVO gave an H_2_/O_2_ ratio of approximately 2:1 and an O_2_ faradaic efficiency of 98.64%, supporting that the measured photocurrent mainly originates from water oxidation (Figure S11).

**FIGURE 3:**
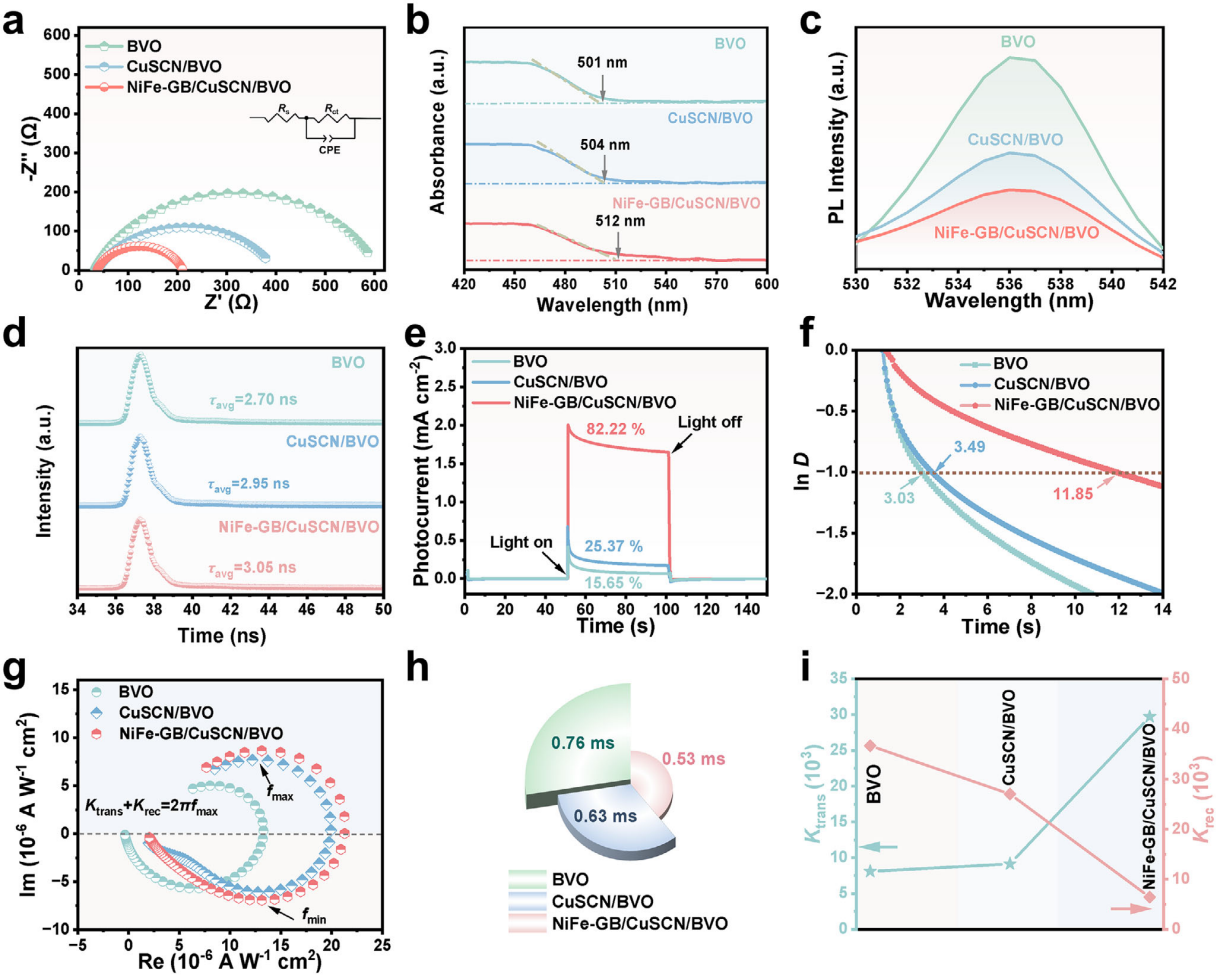
(a) Nyquist plots. (b) UV–vis diffuse reflectance spectra. (c) Steady‐state PL spectra. (d) TRPL decay curves. (e) Transient photocurrent decay spectra. (f) Corresponding ln*D*–t plots derived from transient photocurrent decay analysis. (g) IMPS spectra. (h) *τ*_d_ calculated from IMPS. (i) *K*_trans_ and *K*_rec_ derived from IMPS analysis.

Because photogenerated carrier behavior in PEC systems spans multiple temporal and spatial scales, complementary characterization methods are required to obtain a reliable understanding of charge separation, transfer, and recombination processes [51]. Electrochemical impedance spectroscopy (EIS) measurements reveal a significantly reduction in the charge transfer resistance (*R*_ct_) for the CuSCN/BVO and NiFe‐GB/CuSCN/BVO photoanodes, as evidenced by the markedly smaller semicircles in their Nyquist plots (Figure 3a and Table S3). Furthermore, Mott–Schottky (M–S) analysis reveal *n*‐type semiconductor behavior across all prepared photoanodes, based on the positive slopes observed in the corresponding plots (Figure S12 and Table S4). A negative shift in the flat‐band potential (*E*_flat_) is observed after sequential modification with CuSCN and NiFe‐GB. This shift signifies an upward movement of the Fermi level and a consequent enhancement of band bending at the interface, which promotes the spatial separation of photogenerated charges [52].

UV–vis diffuse reflectance spectra (Figure 3b) show enhanced light absorption upon the introduction of CuSCN and NiFe‐GB, while IPCE results validate that the redshifted absorption is effectively converted into catalytic activity (Figure 2d). The photoluminescence (PL) intensity is progressively quenched following the sequential introduction of CuSCN and NiFe‐GB (Figure 3c), implying reduced radiative recombination of photogenerated carriers. This trend is consistent with the role of CuSCN in extracting holes from BVO and the subsequent consumption of holes by the NiFe‐GB cocatalyst at the surface. From time‐resolved photoluminescence spectroscopy (TRPL) decay (Figure 3d and Table S5), the fitted average lifetime increases slightly from 2.70 ns for pristine BVO to 2.95 ns for CuSCN/BVO and 3.05 ns for NiFe‐GB/CuSCN/BVO. Transient photocurrent decay measurements were further used to evaluate the interfacial charge decay behavior after light interruption (Figure 3e). Compared with pristine BVO, CuSCN/BVO and NiFe‐GB/CuSCN/BVO exhibit slower photocurrent decay, indicating that the extracted photogenerated charges are retained for a longer period and are less prone to rapid recombination. The corresponding normalized ln*D*–t plots show a more gradual decay trend for the modified photoanodes, especially NiFe‐GB/CuSCN/BVO (Figure 3f), further supporting improved charge separation and interfacial charge transfer behavior.

The energy band diagram, derived from Tauc plots and ultraviolet photoelectron spectroscopy (UPS) data (Figures S13–S16), is consistent with the formation of interfacial electronic interaction at the BVO/CuSCN interface. Such an interface may generate a built‐in electric field that is favorable for photogenerated charge separation and hole transport from BVO to CuSCN, thereby mitigating bulk recombination. The intensity‐modulated photocurrent spectroscopy (IMPS) results shown in Figure 3g provide further information on the interfacial charge transfer dynamics. The transient time (*τ*_d_), as determined by equation *τ*_d_ = 1/(2π*f*_min_), shows a decreasing trend from pristine BVO to CuSCN/BVO and then to NiFe‐GB/CuSCN/BVO (Figure 3h and Table S6), indicating that photogenerated carriers require a shorter time to reach the interface. As illustrated in Figure 3i and Table S7, pristine BVO photoanode exhibits an unfavorable kinetic balance, with a relatively high recombination rate constant (*K*_rec_) and low charge transfer rate constant (*K*_trans_), consistent with severe charge recombination. CuSCN modification reduces *K*_rec_, supporting its role in suppressing recombination through hole extraction. After further introducing NiFe‐GB, *K*_trans_ increases and exceeds *K*_rec_ under the tested conditions, indicating that productive hole transfer for water oxidation becomes more favorable than recombination.

To shed light on the distinct role played by the grain boundary microstructure, a control sample was prepared by depositing conventional NiFe layered double hydroxide (LDH) onto CuSCN/BVO via a standard hydrothermal method [53]. The microstructure, elemental composition and distribution, as well as crystal structure of the NiFe LDH/CuSCN/BVO composite were characterized by SEM, TEM, EDS mapping, XRD and XPS survey spectrum, as shown in Figures S17–S20, respectively. The shifts in Fe 2p and Ni 2p binding energies suggest electronic interaction between Ni and Fe in NiFe‐GB/CuSCN/BVO (Figures S21 and S22). Specifically, the localized electron modulation (i.e., Ni→Fe electron flow) enhances the ability of Ni sites to accept nucleophilic species (e.g., OH^−^) generated during water oxidation, while the consequent electron enrichment at Fe sites may influence the adsorption energy of reaction intermediates and contribute to improved OER kinetics [54].

**FIGURE 4:**
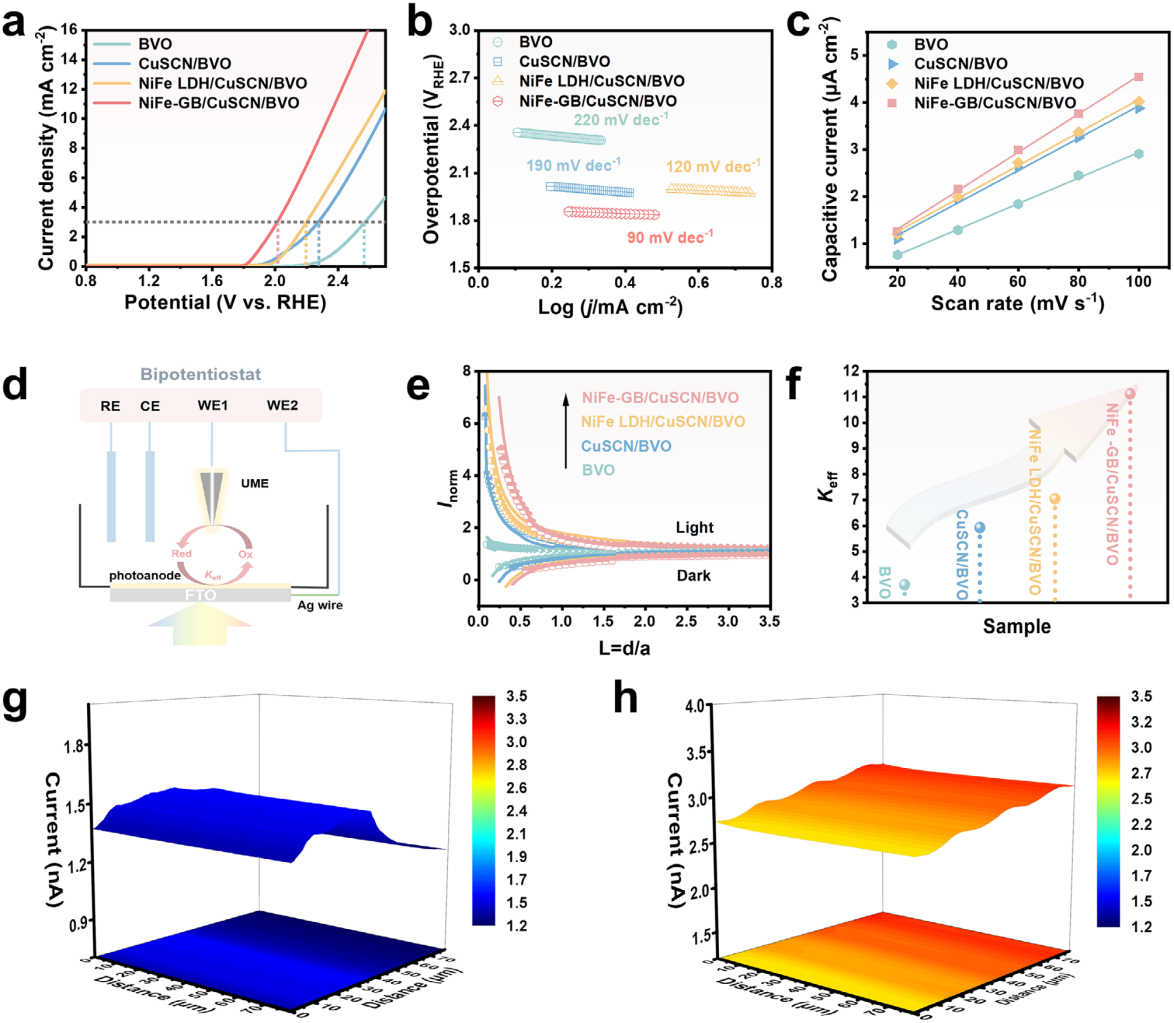
(a) LSV curves measured in the dark. (b) Tafel plots. (c) Double‐layer capacitance (*C*_dl_). (d) Schematic illustration of the feedback‐mode SPECM configuration. (e) Probe approach curves (PACs) and corresponding fitting results. (f) *K*_eff_ derived from SPECM analysis. (g,h) SPECM mapping of the NiFe‐GB/CuSCN/BVO photoanode collected under dark and illuminated conditions.

Furthermore, the NiFe LDH/CuSCN/BVO photoanode delivers a lower photocurrent density of 4.15 mA cm^−2^ at 1.23 V vs. RHE compared with NiFe‐GB/CuSCN/BVO, suggesting the beneficial role of grain‐boundary‐rich NiFe structures (Figure S23). To evaluate the electrocatalytic contribution of different surface modifications, LSV measurements were conducted in the dark. The grain‐boundary‐engineered NiFe‐GB/CuSCN/BVO photoanode shows a lower onset potential and requires a lower potential to reach 3.0 mA cm^−2^ than pristine BVO, CuSCN/BVO, and NiFe LDH/CuSCN/BVO (Figure 4a and Table S8). Notably, the NiFe‐GB/CuSCN/BVO photoanode has a drastically reduced Tafel slope of 90 mV dec^−1^, outperforming other counterparts (Figure 4b and Table S9). The NiFe‐GB/CuSCN/BVO photoanode exhibits the largest double‐layer capacitance (*C*_dl_) derived apparent electrochemically active surface area (ECSA) among the tested samples (Figure 4c, Figures S24 and S25 and Table S10), which may account for its enhanced electrochemical performance. Nevertheless, for semiconductor composite photoanodes, the measured capacitance may also include contributions from surface states, space‐charge capacitance, and pseudocapacitive processes. Therefore, the *C*_dl_‐derived value is discussed here as an apparent indicator rather than a direct measure of the absolute number of active sites.

While the above results support improved charge separation and interfacial charge transfer behavior after NiFe‐GB decoration, spatially resolved evidence is still needed to evaluate whether photogenerated holes can be efficiently delivered to the catalyst/electrolyte interface. Hence, feedback‐mode scanning photoelectrochemical microscopy (SPECM) was performed using a dual working electrode configuration consisting of an ultramicroelectrode (UME) and the photoanode substrate (Figure 4d and Figure S26). The tip‐generated [Fe(CN)_6_]^4−^ diffuses to the illuminated photoanode, where photogenerated holes oxidize it back to [Fe(CN)_6_]^3−^. This regenerative cycle generates a positive feedback current at the tip, providing spatially resolved insights into local photoelectrochemical activity and interfacial charge transfer kinetics at the photoanode surface.

To obtain quantitative kinetic information, probe approach curves (PACs) were collected and fitted using an established theoretical model from prior literature (Figure 4e). The effective heterogeneous charge transfer kinetic constant (*K*_eff_) derived from PAC fitting (Figure 4f and Table S11) follow the order BVO < CuSCN/BVO < NiFe LDH/CuSCN/BVO < NiFe‐GB/CuSCN/BVO. The NiFe‐GB/CuSCN/BVO photoanode exhibits the highest *K*_eff_ value of 11.12 cm s^−1^, corresponding to 3.00‐, 1.88‐, and 1.58‐fold increases relative to pristine BVO, CuSCN/BVO, and NiFe LDH/CuSCN/BVO, respectively. This trend is indicative of more efficient local interfacial charge transfer following the coupling of the CuSCN hole transport layer with the grain‐boundary‐enriched NiFe cocatalyst. Furthermore, the enhanced local photo response observed under illumination, relative to the dark condition, is consistent with the more effective transport of photogenerated holes to the surface, as revealed by the SPECM mapping (Figure 4g,h).

**FIGURE 5:**
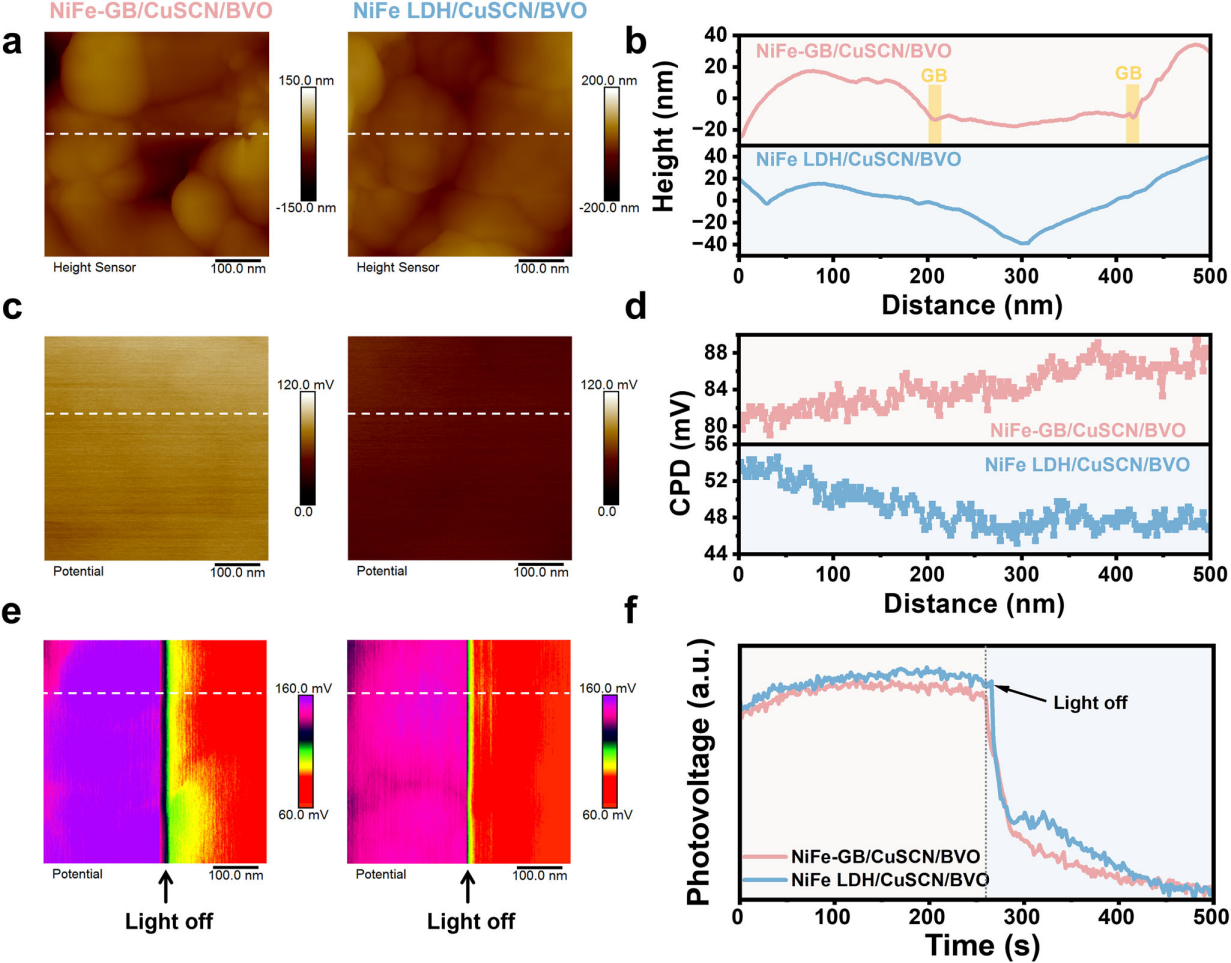
(a) AFM topography of the NiFe‐GB/CuSCN/BVO (left) and NiFe LDH/CuSCN/BVO (right). (b) Height profiles along the scan lines indicated in (a). (c) Corresponding CPD maps. (d) CPD profiles along the scan lines indicated in (c). (e) Relaxation kinetics mapping via KPFM. (f) Relaxation kinetics profiles along the scan lines indicated in (e).

The atomic force microscope (AFM) topography images and corresponding line‐scan profiles (Figure 5a,b) reveal that NiFe‐GB/CuSCN/BVO possesses a lower surface roughness and a higher density of grain boundaries compared to NiFe LDH/CuSCN/BVO. This suggests the absence of significant morphological defects at the grain boundaries and the formation of a more intimate and uniform interfacial contact with CuSCN. Furthermore, the NiFe‐GB/CuSCN/BVO sample exhibits a more negative contact potential difference (CPD), with a mean value of −80.6 vs −46.4 mV for the NiFe LDH/CuSCN/BVO counterpart (Figure 5c), which is consistent with the premise that the grain boundaries facilitate the accumulation of negative charges. Notably, the NiFe‐GB/CuSCN/BVO also shows a smaller ΔCPD, indicating reduced surface potential fluctuations, which is beneficial for more efficient transport of photogenerated carriers (Figure 5d). Kelvin probe force microscopy (KPFM) relaxation kinetics reveal a slower surface photovoltage decay in the NiFe‐GB/CuSCN/BVO photoanode, indicating a prolonged charge‐separated state (Figure 5e,f). This observation supports that the synergistic interface facilitates photogenerated charge separation and interfacial charge accumulation, which is favorable for subsequent hole utilization in water oxidation. This interpretation is also consistent with the slightly prolonged carrier lifetime observed in the TRPL measurements (Figure S27 and Table S5).

**FIGURE 6:**
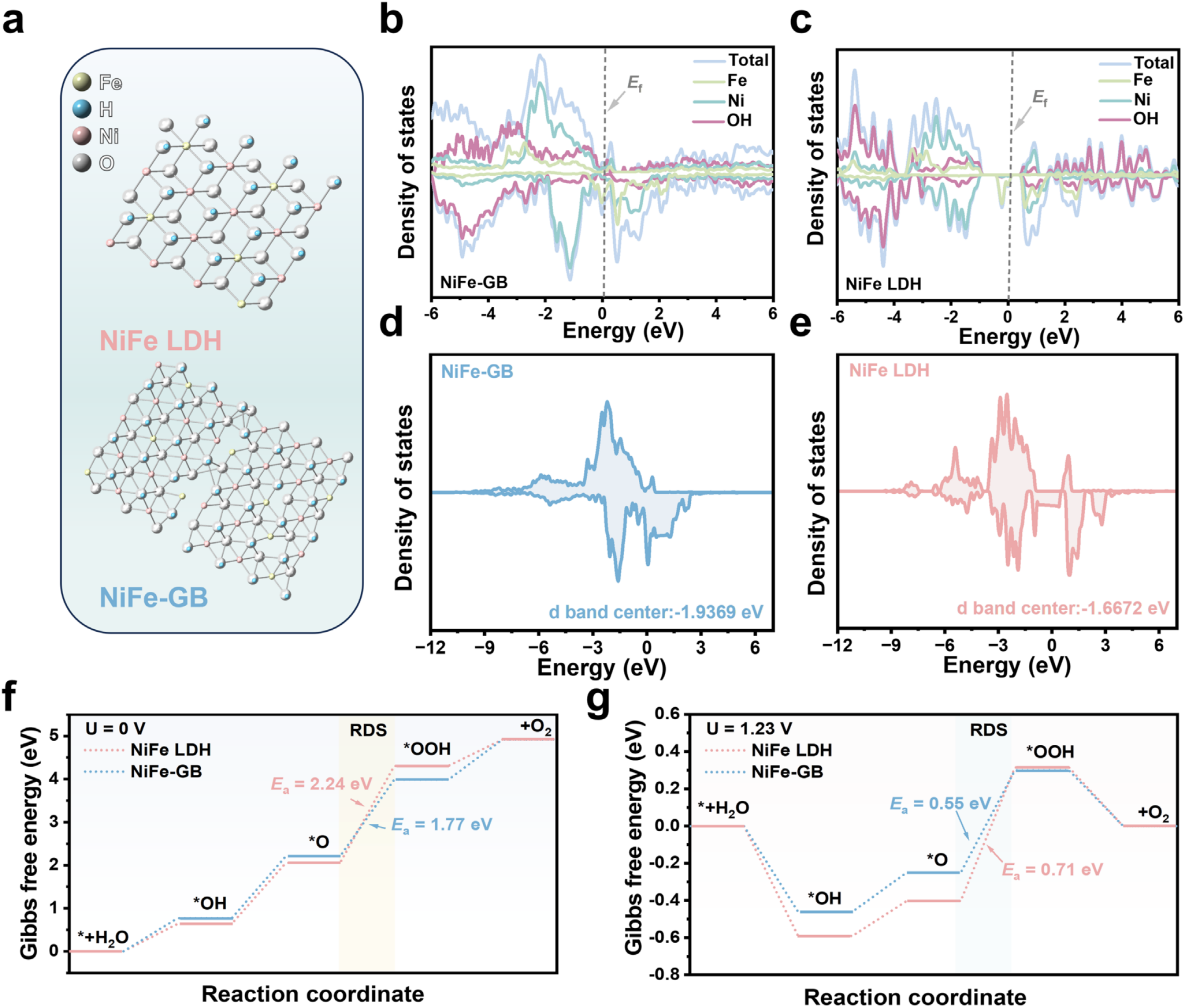
(a) Simplified DFT models of NiFe LDH and NiFe‐GB. (b,c) DOS diagrams of (b) NiFe‐GB and (c) NiFe LDH. (d,e) d‐band centers of (d) NiFe‐GB and (e) NiFe LDH. (f,g) Gibbs free‐energy diagrams for the OER pathway on NiFe LDH and NiFe‐GB at (f) U = 0 V and (g) U = 1.23 V.

To further rationalize how grain‐boundary‐enriched NiFe structures influence OER kinetics, DFT calculations were performed using simplified NiFe‐GB and NiFe LDH models (Figure 6a). It should be noted that the DFT calculations were performed using simplified NiFe‐GB and NiFe LDH models, rather than the full NiFe‐GB/CuSCN/BVO photoelectrode operating in electrolyte under applied bias. Therefore, solvent effects, interfacial electric fields, possible surface reconstruction, and the complete semiconductor/HTL/cocatalyst interface were not explicitly considered. The theoretical results are used here to provide trend‐level mechanistic support for the intrinsic difference between NiFe‐GB and NiFe LDH.

Density of states (DOS) analysis shows that NiFe‐GB possesses a finite total DOS near the Fermi level, whereas NiFe LDH exhibits a much lower DOS around the Fermi level (Figure 6b,c). This trend suggests that the grain‐boundary‐enriched structure may provide more favorable electronic states for interfacial charge transport during OER. In addition, the d‐band center of NiFe‐GB shifts downward relative to that of NiFe LDH (Figure 6d,e), indicating modified metal–adsorbate interactions. Such d‐band modulation may tune the adsorption strength of oxygen‐containing intermediates, preventing excessively strong binding and facilitating subsequent intermediate transformation [55].

The calculated OER free‐energy diagrams further clarify the possible role of grain boundaries in the elementary reaction steps (Figure 6f,g). For both NiFe LDH and NiFe‐GB, the conversion from ^*^O to ^*^OOH (with ^*^ denotes the active site of the catalyst) is identified as the rate‐determining step (RDS). However, the corresponding energy barrier decreases from 2.24 eV for NiFe LDH to 1.77 eV for NiFe‐GB at U = 0 V. At the equilibrium potential of U = 1.23 V, the theoretical overpotential decreases from 0.71 eV for NiFe LDH to 0.55 eV for NiFe‐GB. These results suggest that the grain‐boundary‐enriched NiFe structure may promote OER mainly by modulating intermediate adsorption energetics and lowering the energy barrier for the ^*^O to ^*^OOH transformation, rather than by merely increasing the number of catalytic sites. The trend of above calculations is consistent with the experimentally observed lower onset potential and improved OER kinetics of NiFe‐GB/CuSCN/BVO (Figure 4a).

## 3 Conclusion

By integrating a CuSCN hole transport layer with a grain boundary‐engineered NiFe cocatalyst, this study presents a synergistic strategy that greatly boosts the PEC water oxidation performance of BiVO_4_ photoanodes, affording a high photocurrent density of 6.54 mA cm^−^^2^ at 1.23 V vs. RHE. A stark contrast with conventional layered double hydroxide catalysts underscores the critical contribution of engineered grain boundaries to reaction activity enhancement. Combined experimental results and trend‐level DFT calculations suggest that such boundaries can modulate OER intermediate energetics and lower the energy barrier for the rate‐determining step, thereby contributing to the improved OER kinetics. Overall, this work demonstrates that the coordinated design of charge transport layers and catalyst microstructure can concurrently mitigate bulk/interface charge separation and surface catalysis bottlenecks in photoelectrodes. The strategy offers a generalizable design principle for engineering high‐performance photoelectrodes for efficient solar‐to‐chemical energy conversion.

## Author Contributions

**Wei Zhao**: methodology, data curation, formal analysis, writing – original draft, writing – review and editing, conceptualization. **Yaorong He**: formal analysis, data curation. **Xiao Wang**: data curation. **Lin Zhu**: formal analysis. **Tong Su**: formal analysis. **Yujie Zhou**: data curation. **Xiang Li**: data curation. **Peiyao Du**: conceptualization, data curation, formal analysis, project administration, writing – review and editing, supervision, funding acquisition. **Xiaoquan Lu**: writing – review and editing, formal analysis.

## Funding

National Natural Science Foundation of China (22222408 and 22001193), and the Qin Chuangyuan Innovation and Entrepreneurship Talent Project (No. QCYRCXM‐2022‐338).

## Conflicts of Interest

The authors declare no conflicts of interest.

## Supporting information




**Supporting File**: advs77764‐sup‐0001‐SuppMat.docx.

## Data Availability

The data that support the findings of this study are available from the corresponding author upon reasonable request.
