# Supplementary material for: Grain‐Boundary‐Enriched Cocatalyst Coupled With CuSCN Hole Transport Layer for Efficient Solar Water Oxidation in BiVO4 Photoanode
Source: Adv Sci (Weinh). 2026 Sep 16:e77764. Online ahead of print. doi: 10.1002/advs.77764 (PMC13583217; doi:10.1002/advs.77764)
Supplement: Supplementary file 1 [file ADVS-9999-e77764-s001.docx]

Supporting Information

Grain-Boundary-Enriched Cocatalyst Coupled with CuSCN Hole Transport Layer [for Efficient Solar Water Oxidation in BiVO_4_ Photoanode](https://www.x-mol.com/paperRedirect/1930478830903762944)

Wei Zhao, Yaorong He, Xiao Wang, Lin Zhu, Tong Su, Yujie Zhou, Xiang Li, Peiyao Du,* and Xiaoquan Lu*

**Materials and Methods**

**1. Reagents**

Potassium iodide (KI, anhydrous, AR) was supplied by Sinopharm Group Co., Ltd. Bismuth nitrate pentahydrate (Bi(NO_3_)_3_·5H_2_O, AR), *p*-benzoquinone (C_6_H_4_O_2_, AR), and iron(III) nitrate nonahydrate (Fe(NO_3_)_3_·9H_2_O, 98%) were purchased from [Anhui Zesheng Technology Co., Ltd.](http://www.ciamite.com/Exhibitors-Search/1790.html) Vanadyl acetylacetonate (C_10_H_14_O_5_V, AR), copper(I) thiocyanate (CuSCN, ≥99%), and ammonia solution (NH_3_·H_2_O，28% in H_2_O) were sourced from Shanghai Aladdin Biochemical Technology Co., Ltd. Dimethyl sulfoxide (C_2_H_6_SO, AR), nitric acid (HNO_3_, concentrated, AR), nickel(II) nitrate hexahydrate (Ni(NO_3_)_2_·6H_2_O, AR), and N,N-dimethylformamide (C_3_H_7_NO, AR) were obtained from [Chengdu Chron Chemicals Co,. Ltd](http://chronchem.com/en/#:~:text=%E7%A7%91%E9%9A%86%E5%8C%96%E5%AD%A6%E4%B8%93%E6%B3%A8%E5%90%84%E8%A1%8C%E4%B8%9A%E5%AE%9E%E9%AA%8C%E5%AE%A4%E4%B8%93%E4%B8%9A%E8%AF%95%E5%89%82%E7%A0%94%E5%8F%91%EF%BC%8C%E6%B7%B1%E8%80%95%E5%B8%82%E5%9C%BA%E4%BA%8C%E5%8D%81%E4%BD%99%E5%B9%B4%E3%80%82,%E2%80%9C%E7%A7%91%E8%AF%95%E2%80%9D%E3%80%81%E2%80%9C%E8%AF%BA%E5%B0%94%E6%96%BD%E2%80%9D%E4%B8%A4%E5%A4%A7%E5%93%81%E7%89%8C%E8%83%BD%E6%BB%A1%E8%B6%B3%E6%82%A8%E7%9A%84%E4%B8%AA%E6%80%A7%E5%8C%96%E9%9C%80%E6%B1%82%E3%80%82%20%E5%85%AC%E5%8F%B8%E7%9A%84%E6%B0%A8%E5%9F%BA%E9%85%B8%E8%A1%8D%E7%94%9F%E7%89%A9%E4%BA%A7%E5%93%81%E4%BF%9D%E9%9A%9C%EF%BC%8C%E7%95%85%E9%94%80%E5%9B%BD%E5%86%85%E5%A4%96%E4%B8%BB%E8%A6%81%E5%A4%9A%E8%82%BD%E8%8D%AF%E7%89%A9%E5%88%B6%E9%80%A0%E5%95%86%E3%80%82). Absolute ethanol (C_2_H_6_O, 99.7%) was purchased from Tianjin Tianli Chemical Reagents Co., Ltd. Sodium hydroxide (NaOH, AR) was supplied by Shanghai Titan Scientific Co., Ltd.

**2. Preparation of Photoanodes**

***Preparation of BVO photoanodes***

The synthesis method of the BVO photoanode is consistent with our previous work.^[1]^

***Preparation of*** ***CuSCN/BVO photoanodes***

35 mg of CuSCN powder was dissolved in 2 mL of ammonia solution (NH_3_·H_2_O, 28 wt%) under ultrasonication for 30 min to obtain a homogeneous CuSCN precursor solution. Before CuSCN deposition, the BVO photoanode was rinsed with deionized water and ethanol, dried under N_2_, and fixed on the spin coater. A 100 μL aliquot of the CuSCN precursor solution was dropped onto the BVO surface. The spin-coating process was carried out at 4100 rpm for 30 s. This coating process was repeated three times to form a uniform CuSCN layer, followed by drying at room temperature for 3 h under ambient conditions.

***Preparation of NiFe-GB/CuSCN/BVO photoanodes***

Ni(NO_3_)_2_·6H_2_O (0.232 g) and Fe(NO_3_)_3_·9H_2_O (0.081 g) were dissolved in 5 mL of anhydrous ethanol to form a homogeneous NiFe precursor solution with a Ni/Fe molar ratio of 4:1. The solution was ultrasonicated for 25 min before use. Subsequently, 100 μL of the precursor solution was uniformly dropped onto the surface of a pre-fabricated CuSCN/BVO photoanode, followed by horizontal standing at room temperature for 3 h to allow complete solvent evaporation and precursor deposition. Afterward, the electrode was immersed in ultrapure water for 30 min to induce the formation of the grain-boundary-enriched NiFe hydroxide layer, then rinsed twice with ultrapure water and dried in air at room temperature.

***Preparation of NiFe LDH/CuSCN/BVO photoanodes***

The NiFe LDH/CuSCN/BVO photoanode was synthesized via a combined hydrothermal–solvothermal method.^[2]^ To maintain the same Ni/Fe molar ratio of 4:1 as that used for NiFe-GB/CuSCN/BVO, Ni(NO_3_)_2_·6H_2_O (29.08 mg) and Fe(NO_3_)_3_·9H_2_O (10.1 mg) were dissolved in a mixed solvent containing 30 mL of DMF and 7 mL of deionized water. After ultrasonication until complete dissolution, the homogeneous solution was transferred into a 50 mL Teflon-lined stainless-steel autoclave. The pre-fabricated substrate was placed inside the autoclave with its conductive side facing upward, and the autoclave was heated at 120 °C for 15 h prior to solvothermal treatment at 160 °C for 2 h. After naturally cooling to room temperature, the electrode was thoroughly rinsed with deionized water and dried in air.

**3. Characterization**

X-ray diffraction (XRD) measurements were carried out on a Rigaku SmartLab SE X-ray diffractometer. The morphologies of the as-prepared photoelectrodes were characterized using a scanning electron microscope (SEM, Zeiss Sigma 360) and a transmission electron microscope (TEM, Talos F200X). X-ray photoelectron spectroscopy (XPS, Thermo Scientific K-Alpha) and ultraviolet photoelectron spectroscopy (UPS, Thermo Fisher Scientific ESCALAB Xi+) were used to characterize the chemical composition and valence band of the samples. UV-vis absorption spectra were obtained using a Shimadzu UV-2600i spectrometer, with BaSO_4_ as the reference. Photoluminescence (PL) spectra were measured using a Techcomp FL970 fluorescence spectrophotometer. Time-resolved photoluminescence (TRPL) curves were recorded on a Fluorolog-QM spectrofluorimeter (HORIBA). Atomic force microscope (AFM) measurements were performed on a Bruker ICON system operating in tapping mode in air. A standard OTESPA-R3 probe (Bruker, antimony-doped n-Si, resistivity: 0.01-0.025 Ω·cm) with a resonant frequency of ~300 kHz and a spring constant of 26 N/m was used. Images were acquired at a 256 × 256 pixel resolution and analyzed with NanoScope Analysis software. Gas-evolution measurements were performed on a Pannex A91 Pro gas chromatograph (Pannex Instruments Co., Ltd., Changzhou, China).

**4. Photoelectrochemical (PEC) Measurements**

PEC measurements were carried out with a CHI 760E electrochemical workstation in a standard three-electrode setup, in which the as-fabricated photoanode, an Ag/AgCl electrode, and a Pt electrode acted as the working electrode, reference electrode, and counter electrode, respectively. The photoanode was illuminated by a 300 W Xe lamp (PLS-SXE300E, Perfect Light) equipped with an AM 1.5G filter, with the light intensity calibrated to 100 mW cm⁻^2^ at the WE surface. The electrolyte employed was a 1.0 M potassium borate (KBi) buffer solution (pH 9.5). Linear sweep voltammetry (LSV) was conducted from 0.15 to 1.23 V vs. RHE at a scan rate of 5 mV s^-1^. Electrochemical impedance spectroscopy (EIS) was measured at 0.75 V vs. RHE under AM 1.5 G illumination, with an AC amplitude of 50 mV over a frequency range of 0.1 Hz to 100 kHz. Mott-Schottky (M-S) plots were recorded in the dark at a frequency of 1000 Hz, scanning from -1.0 to 1.0 V vs. Ag/AgCl. Surface charge transfer efficiency (*η*_surface_) was determined in the KBi electrolyte containing 0.2 M Na_2_SO_3_. Intensity-modulated photocurrent spectroscopy (IMPS) and transient photocurrent decay spectra were carried out using a Metrohm Autolab PGSTAT302N electrochemical workstation, with a 450 nm LED source modulated at 10% of its base intensity.

Scanning photoelectrochemical microscopy (SPECM) was performed in feedback mode with a dual working electrode setup, comprising a Pt ultramicroelectrode (tip) and the photoanode (substrate). The electrolyte was a mixed solution of 1 mM K_3_Fe(CN)_6_ and 0.1 M KCl. The substrate and ultramicroelectrode (UME) potentials were maintained at 0.2 and 0 V vs. Ag/AgCl, respectively. The scanning area and step size were 80 × 80 μm^2^ and 1.0 μm, respectively. Probe approach curves were collected at selected regions and fitted using a feedback-mode theoretical model to derive the effective heterogeneous charge transfer kinetic constant (*K*_eff_). All SPECM images compared in this work were acquired under identical instrumental settings and plotted using the same color-scale normalization unless otherwise specified.

The potentials in this study were referenced to the reversible hydrogen electrode (RHE) to enable comparison with the redox potentials of H_2_ and O_2_. Potentials vs. Ag/AgCl were converted to the RHE scale via the Nernst equation:

$E_{RHE}=E_{Ag/AgCl}^{applied}+0.0591\times pH+E_{Ag/AgCl}^{0}$	**eqn. S1**

Surface charge transfer efficiency (*η*_surface_) and bulk charge seperation efficiency (*η*_bulk_) can be calculated by following equations:

$\eta_{\mathrm{surface}}=\frac{J_{H_{2}O}}{J_{\mathrm{Na}_{2}\mathrm{SO}_{3}}}$	**eqn. S2**

$\eta_{\mathrm{bulk}}=\frac{J_{\mathrm{Na}_{2}\mathrm{SO}_{3}}}{J_{\mathrm{abs}}}$	**eqn. S3**

Incident photon-to-current efficiency (IPCE) can be calculated using the following formula:

$IPCE=\frac{J\times1240}{\lambda\times P_{\lambda}}\times100\%$  **eqn. S4**

*J* represents the photocurrent density (mA/cm^2^). *λ* denotes the incident monochromatic light wavelength (nm), while *P*_λ_ is the power density (mW/cm^2^) measured at a specific wavelength.

Applied bias photon-to-current efficiency (ABPE) can be calculated using the following formula:

$ABPE=\frac{J\times\left( 1.23-V_{\mathrm{applied}} \right)}{P_{\mathrm{total}}}$  **eqn. S5**

*V*_applied_ is the applied bias vs. RHE (V), and *P*_total_ is the total light intensity of AM 1.5 G (100 mW/cm^2^).

Time-resolved photoluminescence (TRPL) data can be well fitted by a bi-exponential function of time (t):

$I\left( t \right)=A_{1}e^{-\frac{1}{\tau_{1}}}+A_{2}e^{-\frac{1}{\tau_{2}}}+I_{0}$	**eqn. S6**

where *τ*_1_ and *τ*_2_ refer to the time constants of the fast and slow decay processes, respectively.

Based on the fitting TRPL results, the average recombination lifetime (*τ*_avg_) can be calculated as follows:

$\tau_{avg}=\frac{\left( A_{1}\tau_{1}^{2}+A_{2}\tau_{2}^{2} \right)}{\left( A_{1}\tau_{1}+A_{2}\tau_{2} \right)}$ **eqn. S7**

Defining the lowest point of the high-frequency portion in IMPS as *f*_min_, the transient time (*τ*_d_) is obtainable via the following equation:

$\tau_{d}=\frac{1}{2\pi f_{\min}}$	**eqn. S8**

Transient photocurrents (TP) were analyzed by converting them into a normalized parameter *D*, calculated as:

$D=\frac{J_{t}-J_{st}}{J_{in}-J_{st}}$	**eqn. S9**

where *J*_t_, *J*_st,_ and *J*_in_ are the time-dependent, steady-state, and instantaneous photocurrent densities, respectively.

Charge transfer (*K*_trans_) and recombination (*K*_rec_) rate constants were estimated via the following equation:

$\frac{J_{st}}{J_{in}}=\frac{K_{trans}}{K_{rec}+K_{trans}}$	**eqn. S10**

**5. Density Functional Theory (DFT) Calculations**

The calculations were based on density functional theory (DFT) as implemented in the Vienna Ab Initio Simulation Package (VASP). The exchange-correlation effects were described using the generalized gradient approximation (GGA) with the Perdew-Burke-Ernzerhof (PBE) functional.^[3–5]^ The projector augmented wave (PAW) method and a plane-wave basis set with an energy cutoff of 500 eV were used to describe the ionic cores and valence electrons, respectively.^[6,7]^ Fractional occupancies of the Kohn-Sham orbitals were treated with the Gaussian smearing method (width = 0.05 eV). The electronic and geometric relaxations were considered converged when the energy change between successive steps fell below 10⁻^5^ eV and the maximum force on any atom was less than 0.05 eV/Å, respectively. Dispersion interactions were modeled via Grimme's DFT-D3 correction.^[8]^ A vacuum layer of 20 Å was used in the direction normal to the surface to avoid spurious interactions. For the Brillouin zone integration, a 2×2×1 Monkhorst-Pack k-point grid was used for the surface structure. Finally, the adsorption energies (*E*_ads_) were calculated as *E*_ads_ = *E*_ad/sub_ -*E*_ad_ -*E*_sub_, where *E*_ad/sub_, *E*_ad_, and *E*_sub_ are the total energies of the optimized adsorbate/substrate system, the adsorbate in the structure, and the clean substrate, respectively. The Gibbs free energy of reaction (G) was calculated using the equation:

G= *E*_ads_ +ZPE-TS

where G, *E*_ads_, ZPE and TS are the free energy, total energy from DFT calculations, zero point energy, and entropic contributions, respectively.

**Results and Discussion**

**Figure S1.** Local magnification of HR-TEM image of NiFe-GB/CuSCN/BVO.

**Figure S2.** X-ray diffraction (XRD) patterns of BVO, CuSCN/BVO, and NiFe-GB/CuSCN/BVO.

**Figure S3.** Raman spectrum of BVO, CuSCN/BVO, and NiFe-GB/CuSCN/BVO.

**Figure S4.** Full XPS survey spectrum of the NiFe-GB/CuSCN/BVO photoanode.

**Figure S5.** (a) Bi 4f, (b) O 1s, and (c) V 2p XPS spectra of BVO, CuSCN/BVO, and NiFe-GB/CuSCN/BVO. (d) Cu 2p XPS spectra of CuSCN/BVO and NiFe-GB/CuSCN/BVO. (e) Fe 2p and (f) Ni 2p XPS spectra of NiFe-GB/CuSCN/BVO.

**Figure S6.** N1s XPS spectra of CuSCN/BVO and NiFe-GB/CuSCN/BVO.

**Figure S7.** Multiple independent LSV measurements of photocurrent density for independently prepared (a) BVO, (b) CuSCN/BVO, and (c) NiFe-GB/CuSCN/BVO photoanodes under AM 1.5G illumination (n = 5).

**Figure S8.** LSV curves measured in an electrolyte with 0.2 M Na_2_SO_3_ as the hole scavenger.

**Figure S9.** SEM image of NiFe-GB/CuSCN/BVO after the stability test.

**Figure S10.** XRD pattern of NiFe-GB/CuSCN/BVO after the stability test.

**Figure S11.** Time-dependent gas evolution of H_2_ and O_2_ over NiFe-GB/CuSCN/BVO during PEC water splitting under AM 1.5G illumination.

**Figure S12.** Mott-Schottky plots of BVO, CuSCN/BVO, and NiFe-GB/CuSCN/BVO.

**Figure S13.** Band diagram of NiFe-GB/CuSCN/BVO.

**Figure S14.** Tauc plots of BVO, CuSCN/BVO, and NiFe-GB/CuSCN/BVO.

**Figure S15.** UV-vis absorption spectra (a) and Tauc plot (b) of CuSCN.

**Figure S16.** Ultraviolet photoelectron spectroscopy (UPS).

The band edges of BVO relative to the vacuum level are positioned at -7.81eV (VBM) and -5.39 eV (CBM), calculated from work function of 4.24 eV (*E*_F_ = -4.24 eV), VBM binding energy offset of 3.57 eV, and an optical bandgap of 2.42 eV. Likewise, for CuSCN, the corresponding band edges are located at -7.51eV (VBM) and -3.95 eV (CBM), derived from work function of 4.84 eV (*E*_F_ = -4.84 eV), VBM binding energy offset of 2.67 eV, and an optical bandgap of 3.56 eV. These calculations establish a band alignment between BVO and CuSCN, which facilitates efficient charge separation in heterojunction. The Fermi level of BVO (-4.24 eV) is higher than that of CuSCN (-4.84 eV). Upon contact, electrons flow from BVO to CuSCN until their Fermi levels align, resulting in the formation of a built-in electric field at the interface that promotes charge separation.

**Figure S17.** SEM image of NiFe LDH/CuSCN/BVO.

**Figure S18.** (a) TEM image, (b) HR-TEM image and (c) EDS element mappings of Bi, O, V, Fe, Ni, and Cu of NiFe LDH/CuSCN/BVO.

TEM analysis confirms the absence of abundant grain boundaries within the NiFe-LDH material, and the successful synthesis of the NiFe LDH/CuSCN/BVO composite is further verified by EDS elemental mapping.

**Figure S19.** XRD pattern of NiFe LDH/CuSCN/BVO.

**Figure S20.** Full XPS survey spectrum of NiFe LDH/CuSCN/BVO.

**Figure S21.** Fe 2p peaks in the high-resolution XPS spectrum of NiFe LDH/CuSCN/BVO.

**Figure** **S22.** Ni 2p peaks in the high-resolution XPS spectrum of NiFe LDH/CuSCN/BVO.

**Figure S23.** LSV of NiFe LDH/CuSCN/BVO.

**Figure S24.** CV curves of BVO, CuSCN/BVO, NiFe LDH/CuSCN/BVO, and NiFe-GB/CuSCN/BVO photoanodes at different scan rates.

**Figure S25.** Electrochemically active surface area (ECSA) of BVO, CuSCN/BVO, NiFe LDH/CuSCN/BVO, and NiFe-GB/CuSCN/BVO.

**Figure S26.** (a) SPECM setup; (b) Photo of UME.

**Figure S27.** Time-resolved photoluminescence (TRPL) spectra of the as-prepared samples.

**Table S1.** Comparison of the PEC performance of NiFe-GB/CuSCN/BVO with representative BVO-based photoanodes.

| **Photoanode** | ***J* (mA cm^-2^) at 1.23 V_RHE_** | **Electrolyte solution** | **Ref.** |
| --- | --- | --- | --- |
| BiVO_4_/PEI/TiO_2_/CoOOH | 2.03 | 0.5 M Potassium Phosphate Buffer (pH = 8.0) | ^[9]^ |
| BiVO_4_/BiO_x_/NiFeO_x_ | 6.34 | 1.0 M Potassium Borate Solution (pH = 9.3) | ^[10]^ |
| BiVO_4_@NM88B(Fe)/Co_4_O_4_ | 5.26 | 0.5 M Potassium Borate Solution (pH = 9.31) | ^[11]^ |
| FeOOH/Ni-N_4_-O/BiVO_4_ | 6.0 | 0.5 M Potassium Borate Solution (pH = 9.3) | ^[12]^ |
| O_v_-BiVO_4_/MIL-101 | 5.91 | 1.0 M Potassium Borate Solution (pH = 9.0) | ^[13]^ |
| BiVO_4_-NLs/WO_3_-NRs | 2.83 | 0.5 M Potassium Phosphate Buffer (pH = 7.3) | ^[14]^ |
| VMo-BiVO_4_ | 3.18 | 0.2 M Potassium Phosphate Buffer (pH = 7.0) | ^[15]^ |
| BVO@ZCF(P)-O | 5.7 | 1.0 M Potassium Borate Solution (pH = 9.0) | ^[16]^ |
| Cu-RuO_2_-Tm-BiVO_4_ | 5.3 | 0.5 M Sodium Sulfate Solution (pH = 6.4) | ^[17]^ |
| SMSI-CoPi/Au/BiVO_4_ | 5.01 | 0.5 M Potassium Borate Solution (pH = 9.3) | ^[18]^ |
| BiVO_4_/N:NiFeO_x_ | 6.4 | 0.5 M Potassium Borate Solution (pH = 9.5) | ^[19]^ |
| PHBVO | 6.36 | 1.0 M Potassium Borate Solution (pH = 9.5) | ^[20]^ |
| NiFeOₓ/H-BiVO₄ | 4.68 | 1.0 M Potassium Borate Solution (pH = 9.5) | ^[21]^ |
| FeCoNiMoCrOOH/BiVO_4_ | 5.23 | 0.5 M Potassium Borate Solution (pH = 9.5) | ^[22]^ |
| Co_3_O_4_/CoV-MOF/BiVO_4_ | 6.0 | 1 M Potassium Borate Solution (pH = 9.5) | ^[23]^ |
| ε-FeOOH/BiVO_4_ | 5.4 | 0.2 M Sodium Sulfate Solution (pH = 7.0) | ^[24]^ |
| H-CoAl-LDH/BiVO_4_ | 3.5 | 0.5 M Sodium Sulfate Solution (pH = 7.35) | ^[25]^ |
| PTh-Fe/BVO | 4.72 | 0.5 M Potassium Borate Solution (pH = 9.0) | ^[26]^ |
| AuCo(OH)_x_/BiVO_4_ | 6.2 | 0.5 M Potassium Borate Solution (pH = 9.5) | ^[27]^ |
| BiVO_4_/Co-TCPP-FAA | 5.47 | 1.0 M Potassium Tetraborate Solution (pH = 9.5) | ^[28]^ |
| NiFe-GB/CuSCN/BVO | 6.54 | 1.0 M Potassium Borate Solution (pH = 9.5) | This work |

**Table S2.** Photocurrent intensities derived from multiple independent measurements.

| **Sample** | **J (mA cm^-2^) at 1.23 V vs. RHE** | | | | |
| --- | --- | --- | --- | --- | --- |
| BVO | 1.79 | 1.83 | 1.90 | 1.95 | 2.10 |
| CuSCN/BVO | 3.35 | 3.36 | 3.41 | 3.46 | 3.52 |
| NiFe-GB/CuSCN/BVO | 6.39 | 6.54 | 6.43 | 6.49 | 6.51 |

**Table S3.** Fitted *R*_s_ and *R*_ct_ values of BVO-based photoanodes obtained from EIS spectra.

| **Sample** | ***R*_s_ (Ω)** | ***R*_ct_ (Ω)** |
| --- | --- | --- |
| BVO | 32.36 | 574.6 |
| CuSCN/BVO | 35.77 | 362.6 |
| NiFe-GB/CuSCN/BVO | 37.31 | 185.8 |

**Table S4.** Charge carrier density values of BVO-based photoanodes obtained from M-S plots.

| **Sample** | **Charge carrier density (×10^18^ cm^-3^)** |
| --- | --- |
| BVO | 2.40 |
| CuSCN/BVO | 3.13 |
| NiFe-GB/CuSCN/BVO | 3.92 |

**Table S5.** Fitted results (carrier lifetime) from the time-resolved photoluminescence spectroscopy.

| **Sample** | ***τ*_1_ (ns)** | **A_1_ (%)** | ***τ*_2_ (ns)** | **A_2_ (%)** | ***τ*_avg_ (ns)** |
| --- | --- | --- | --- | --- | --- |
| BVO | 2.50 | 76.22 | 13.11 | 2.04 | 2.70 |
| CuSCN/BVO | 2.31 | 24.20 | 13.00 | 1.55 | 2.95 |
| NiFe LDH/CuSCN/BVO | 2.09 | 12.16 | 13.31 | 1.02 | 2.96 |
| NiFe-GB/CuSCN/BVO | 2.58 | 29.06 | 13.73 | 1.35 | 3.05 |

**Table S6.** Transient time values for all photoanodes obtained from IMPS spectra.

| **Sample** | ***f*_min_ (Hz)** | ***τ*_d_ (ms)** |
| --- | --- | --- |
| BVO | 208.93 | 0.76 |
| CuSCN/BVO | 251.19 | 0.63 |
| NiFe-GB/CuSCN/BVO | 302.00 | 0.53 |

**Table S7.** Charge transfer rate constant (*K*_trans_) and recombination rate constant (*K*_rec_) values of pristine and modified photoanode systems.

| **Sample** | ***K*_trans_ (10^3^ s^-1^)** | ***K*_rec_ (10^3^ s^-1^)** |
| --- | --- | --- |
| BVO | 8.09 | 36.65 |
| CuSCN/BVO | 9.13 | 27.01 |
| NiFe-GB/CuSCN/BVO | 29.71 | 6.42 |

**Table S8.** Potentials required to reach a current density of 3.0 mA cm^−2^.

| **Sample** | **Potential (V vs. RHE) at 3.0 mA cm^-2^** |
| --- | --- |
| BVO | 2.56 |
| CuSCN/BVO | 2.28 |
| NiFe LDH/CuSCN/BVO | 2.20 |
| NiFe-GB/CuSCN/BVO | 2.01 |

**Table S9.** Tafel slopes of BVO-based photoanodes.

| **Sample** | **Tafel slope (mV dec^-1^)** |
| --- | --- |
| BVO | 220 |
| CuSCN/BVO | 190 |
| NiFe LDH/CuSCN/BVO | 120 |
| NiFe-GB/CuSCN/BVO | 90 |

**Table S10.** Electrochemical surface area (ECSA) values of the pristine and modified photoanode samples.

| **Sample** | **ECSA (cm^2^)** |
| --- | --- |
| BVO | 0.50 |
| CuSCN/BVO | 0.73 |
| NiFe LDH/CuSCN/BVO | 0.75 |
| NiFe-GB/CuSCN/BVO | 1.25 |

**Table S11.** *K*_eff_ values derived from SPECM measurements.

| **Sample** | ***K*_eff_ (cm s^-1^)** |
| --- | --- |
| BVO | 3.71 |
| CuSCN/BVO | 5.93 |
| NiFe LDH/CuSCN/BVO | 7.05 |
| NiFe-GB/CuSCN/BVO | 11.12 |

**References**

[1]	W. Zhao, Z. Wang, H. Huang, Y. He, S. Zou, L. Zhu, H. Xiao, X. Ning, W. Luo, P. Du, X. Lu, “Unveiling fast charge transfer dynamics at semiconductor/electrocatalyst/electrolyte dual interfaces via boron engineering for efficient water splitting” *Adv. Energy Mater.* **2025**, *15*, e04275.

https://doi.org/10.1002/aenm.202504275

[2]	L. Wang, F. Dionigi, N. T. Nguyen, R. Kirchgeorg, M. Gliech, S. Grigorescu, P. Strasser, P. Schmuki, “Tantalum nitride nanorod arrays: introducing Ni–Fe layered double hydroxides as a cocatalyst strongly stabilizing photoanodes in water splitting” *Chem. Mater.* **2015**, *27*, 2360–2366.

https://doi.org/10.1021/cm503887t

[3]	J. P. Perdew, K. Burke, M. Ernzerhof, “Generalized gradient approximation made simple” *Phys. Rev. Lett.* **1996**, *77*, 3865–3868.

https://doi.org/10.1103/PhysRevLett.77.3865

[4]	G. Kresse, J. Furthmüller, “Efficient iterative schemes for *ab initio* total-energy calculations using a plane-wave basis set” *Phys. Rev. B* **1996**, *54*, 11169–11186.

https://doi.org/10.1103/PhysRevB.54.11169

[5]	G. Kresse, D. Joubert, “From ultrasoft pseudopotentials to the projector augmented-wave method” *Phys. Rev. B* **1999**, *59*, 1758–1775.

https://doi.org/10.1103/PhysRevB.59.1758

[6]	S. Grimme, J. Antony, S. Ehrlich, H. Krieg, “A consistent and accurate *ab initio* parametrization of density functional dispersion correction (DFT-D) for the 94 elements H-Pu” *J. Chem. Phys.* **2010**, *132*, 154104.

https://doi.org/10.1063/1.3382344

[7]	P. E. Blöchl, “Projector augmented-wave method” *Phys. Rev. B* **1994**, *50*, 17953–17979.

https://doi.org/10.1103/PhysRevB.50.17953

[8]	G. Henkelman, B. P. Uberuaga, H. Jónsson, “A climbing image nudged elastic band method for finding saddle points and minimum energy paths” *J. Chem. Phys.* **2000**, *113*, 9901–9904.

https://doi.org/10.1063/1.1329672

[9]	S. Bae, T. Moehl, E. Service, M. Kim, P. Adams, Z. Wang, Y. Choi, J. Ryu, S. D. Tilley, “A hole-selective hybrid TiO_2_ layer for stable and low-cost photoanodes in solar water oxidation” *Nat. Commun.* **2024**, *15*, 9439.

https://doi.org/10.1038/s41467-024-53754-9

[10]	N. Li, Y. Wei, S. Liu, Z. Yu, Y. Shen, M. Wang, “Boosting oxygen evolution reaction performance on BiVO_4_ photoanode via gradient oxygen vacancies” *ACS Energy Lett.* **2025**, *10*, 2162–2170.

https://doi.org/10.1021/acsenergylett.5c00507

[11]	H. Li, Z. Hou, Y. Hu, J. Wang, A. Li, P. F.-X. Corvini, “Covalent bonding mediated interfacial charge transfer between BiVO_4_ and {Co_4_O_4_} cubanes for enhanced photoelectrochemical water splitting” *Adv. Funct. Mater.* **2026**, *36*, e23057.

https://doi.org/10.1002/adfm.202523057

[12]	X. Zhang, P. Zhai, Y. Zhang, Y. Wu, C. Wang, L. Ran, J. Gao, Z. Li, B. Zhang, Z. Fan, L. Sun, J. Hou, “Engineering single-atomic Ni-N_4_-O sites on semiconductor photoanodes for high-performance photoelectrochemical water splitting” *J. Am. Chem. Soc.* **2021**, *143*, 20657–20669.

https://doi.org/10.1021/jacs.1c07391

[13]	Y. Xin, J. Tian, X. Xiong, C. Wu, S. A.C. Carabineiro, X. Yang, Z. Chen, Y. Xia, Y. Jin, “Enhanced photocatalytic efficiency through oxygen vacancy‐driven molecular epitaxial growth of metal–organic frameworks on BiVO_4_” *Adv. Mater.* **2025**, *37*, 2417589.

https://doi.org/10.1002/adma.202417589

[14]	J.-H. Kim, J. W. Yoon, T.-H. Kim, Y.-M. Jo, J.-S. Kim, S.-Y. Jeong, J.-H. Lee, “Heterostructure between WO_3_ and metal organic framework-derived BiVO_4_ nanoleaves for enhanced photoelectrochemical performances” *Chem. Eng. J.* **2021**, *425*, 131496.

https://doi.org/10.1016/j.cej.2021.131496

[15]	X. Yin, W. Qiu, W. Li, C. Li, K. Wang, X. Yang, L. Du, Y. Liu, J. Li, “High porosity Mo doped BiVO_4_ film by vanadium re-substitution for efficient photoelectrochemical water splitting” *Chem. Eng. J.* **2020**, *389*, 124365.

https://doi.org/10.1016/j.cej.2020.124365

[16]	J.-B. Pan, B.-H. Wang, S. Shen, L. Chen, S.-F. Yin, “Introducing bidirectional axial coordination into BiVO_4_@metal phthalocyanine core–shell photoanodes for efficient water oxidation” *Angew. Chem. Int. Ed.* **2023**, *62*, e202307246.

https://doi.org/10.1002/anie.202307246

[17]	K. Tian, L. Jin, A. Mahmood, H. Yang, P. An, J. Zhang, Y. Ji, Y. Li, D. Li, S. (Frank) Liu, J. Yan, “Lattice distortion promotes carrier separation to improve the photoelectrochemical water splitting performance of bismuth vanadate photoanode” *Adv. Funct. Mater.* **2024**, *34*, 2410548.

https://doi.org/10.1002/adfm.202410548

[18]	Y. Cao, Y. Tian, B. He, Z. Qiao, L. Li, Y. Zhu, Y. Yang, X. Liu, “Modulating hole extraction and water oxidation kinetics in CoPi/Au/BiVO_4_ photoanode via strong metal-support interactions” *J. Energy Chem.* **2025**, *109*, 315–324.

https://doi.org/10.1016/j.jechem.2025.05.048

[19]	B. Zhang, S. Yu, Y. Dai, X. Huang, L. Chou, G. Lu, G. Dong, Y. Bi, “Nitrogen-incorporation activates NiFeO_x_ catalysts for efficiently boosting oxygen evolution activity and stability of BiVO_4_ photoanodes” *Nat. Commun.* **2021**, *12*, 6969.

https://doi.org/10.1038/s41467-021-27299-0

[20]	S. Wang, H. Yuan, J. Feng, L. Xu, M. Guo, W. Huang, S. Wang, “Phosphorous-doped high-entropy oxides enabling full spectrum utilization of BiVO_4_ photoanodes for efficient water oxidation” *Adv. Funct. Mater.* **2026**, *36*, e12757.

https://doi.org/10.1002/adfm.202512757

[21]	H. Wu, L. Zhang, S. Qu, A. Du, J. Tang, Y. H. Ng, “Polaron-mediated transport in BiVO_4_ photoanodes for solar water oxidation” *ACS Energy Lett.* **2023**, *8*, 2177–2184.

https://doi.org/10.1021/acsenergylett.3c00465

[22]	L. Wang, Z. Gao, K. Su, N. T. Nguyen, R.-T. Gao, J. Chen, L. Wang, “Stacked high-entropy hydroxides promote charge transfer kinetics for photoelectrochemical water splitting” *Adv. Funct. Mater.* **2024**, *34*, 2403948.

https://doi.org/10.1002/adfm.202403948

[23]	L. Xu, Y. Zhang, B. Liu, K. Wan, X. Wang, T. Wang, L. Wang, S. Wang, W. Huang, “Strengthening bonding interaction of a (Co_0.91_V_0.09_)_3_(BTC)_2_ metal–organic framework with BiVO_4_ photoanodes enabling ultrastable photoelectrochemical water oxidation” *ACS Nano* **2025**, *19*, 15863–15875.

https://doi.org/10.1021/acsnano.5c01111

[24]	Y.-F. Hu, Y.-F. Li, Z.-P. Liu, “Structural origin for efficient photoelectrochemical water splitting over Fe-modified BiVO_4_” *ACS Catal.* **2023**, *13*, 10167–10176.

https://doi.org/10.1021/acscatal.3c02504

[25]	P. Yue, H. She, L. Zhang, B. Niu, R. Lian, J. Huang, L. Wang, Q. Wang, “Super-hydrophilic CoAl-LDH on BiVO_4_ for enhanced photoelectrochemical water oxidation activity” *Appl. Catal., B* **2021**, *286*, 119875.

https://doi.org/10.1016/j.apcatb.2021.119875

[26]	J. Miao, C. Lin, X. Yuan, Y. An, Y. Yang, Z. Li, K. Zhang, “Supramolecular catalyst with [FeCl_4_] unit boosting photoelectrochemical seawater splitting via water nucleophilic attack pathway” *Nat. Commun.* **2024**, *15*, 2023.

https://doi.org/10.1038/s41467-024-46342-4

[27]	X. Chen, X. Li, Y. Peng, H. Yang, Y. Tong, M.-S. Balogun, Y. Huang, “Tailoring carrier dynamics of BiVO_4_ photoanode via dual incorporation of Au and Co(OH)_x_ cooperative modification for photoelectrochemical water splitting” *Adv. Funct. Mater.* **2025**, 35, 2416091.

https://doi.org/10.1002/adfm.202416091

[28]	C. Zhang, Y. Wang, W. Sun, Z. Hua, Z. Zhang, S. Gong, D. Wang, Y. Tian, “Tailoring non-covalent interaction via single atom to boost interfacial charge transfer toward photoelectrochemical water oxidation” *Adv. Mater.* **2024**, 37, 2410632.

https://doi.org/10.1002/adma.202410632
